# Esthetic and functional reconstruction of large mandibular defects using free fibula flap and implant-retained prosthetics - a case series with long-term follow-up

**DOI:** 10.1186/s13005-021-00297-9

**Published:** 2021-10-20

**Authors:** Tamás Sass, József Piffkó, Gábor Braunitzer, Ferenc Oberna

**Affiliations:** 1 Department of Oral and Maxillofacial Surgery and Otolaryngology, Bács-Kiskun County Hospital, Teaching Hospital of the University of Szeged, Nyíri út 38, Kecskemét, Hungary; 2 University of Szeged, Doctoral School of Clinical Medicine, 6720 Szeged, Korányi fasor 6., Szeged, Hungary; 3 Department of Oral and Maxillofacial Surgery, Faculty of Medicine, University of Szeged, Kálvária sugárút 57, Szeged, Hungary; 4 Chief researcher, dicomLAB Dental Ltd., Szent-György Albert utca 2, Szeged, Hungary; 5 Multidisciplinary Head and Neck Cancer Center, National Institute of Oncology, Ráth György utca 7-9, Budapest, Hungary

**Keywords:** Mandibular defect, Reconstructive surgery, Free fibula flap, Implant-retained prosthetics, Case report

## Abstract

**Background:**

The reconstructive and rehabilitative management of large mandibular defects with basal continuity is challenging in many respects, especially in the vertical dimension. The free fibula flap is an under-utilised but efficient approach in this indication. The aim of this case series is to demonstrate its use and long-term success.

**Case presentation:**

Three cases are presented, where the patient had a large bone defect (at least 5 cm in length and 1 cm in the vertical dimension), but the continuity of the mandible was maintained. Two cases were related to pathological fracture and one was a large defect due to oncological surgery. Vertical augmentation with free microvascularised fibula flap was carried out, followed by implant-retained prosthetic therapy. Clinical status has been followed up for 5 to 6 years, with special attention to the condition of the peri-implant tissues and any radiographically detectable alterations or complications. No complications occurred during the follow-up. Function and esthetics have remained unchanged throughout.

**Conclusions:**

Free microvascularised fibula flap reconstruction combined with implant-retained prosthetics allows a lasting functional and esthetic solution in the discussed indication.

## Background

Today, bone augmentation procedures in dental rehabilitation are considered a standard element of dental care, especially given the increased availability of modern osteoplastic materials. Bone grafting methods are versatile both in terms of their indication and their technical aspects [1, 2]. Still, autologous bone augmentation remains the gold standard, especially in difficult cases, as it allows for the transplantation of cells capable of osteogenesis [3]. Lateral augmentation performed prior to implantation is safe and reliable. However, the reconstruction of large vertical bone defects, especially in the mandible, remains a major clinical challenge [1, 2]. This is explained by the fact that the volume of augmented bone is more likely to collapse and that during vertical augmentation, creating a tension-free soft tissue closure is difficult to achieve [4].

Our maxillofacial department has performed maxillofacial reconstructive procedures for over 10 years. During this period, 460 microsurgical procedures have been accomplished. Of these, we present three rarely seen cases, where the patient had a large bone defect (at least 5 cm in length and 1 cm in the vertical dimension), but the continuity of the mandible was maintained, nevertheless. In all cases, it was possible to resolve the situation with vertical augmentation in a way that allowed esthetic, implant-based prosthetic treatment. Such defects are especially difficult to manage, because, without the application of microvascularised free flap, there is a high risk of significant bone absorption. Also, it is extremely difficult to provide tension-free soft tissue covering over such large augmented areas.

Given that these lesions are rare, it comes as no surprise that little is to be found in the literature about their treatment. With the presented case series, we sought to share our experience and demonstrate that autologous vertical augmentation with free microvascularised flap is a reliable method to treat such cases in a functionally and esthetically favorable manner.

This study was approved by the Institutional Review Board (Nos. 35/2005. (VIII.26) and 3/17.01.25.). Written informed consent was obtained from all patients before each procedure described in this report. The patients gave their written consent retrospectively to their data being included in this study. The patients gave written consent to the publishing of their images.

## Case presentation

### Shared characteristics and procedures

The three cases shared the following characteristics: the mandibular continuity was maintained with a vertical bone defect of at least 1 cm over a segment of at least 5 cm, and the distance between the inferior alveolar nerve or the base of the mandible and the alveolar ridge was smaller than 5 mm.

Two cases were due to extreme mandibular atrophy and pathological fracture, and in one case, the reconstruction of the height of the mandible was necessitated by a previous oncological surgery.

Regardless of the underlying cause, our objective with the reconstruction of the alveolar process was making aesthetic implant treatment possible and creating keratinised gingiva where possible. The latter is a basic prerequisite of long - term success in implant prosthetics [5–8]. In all presented cases, autologous vertical augmentation with free microvascularised flap was utilised.

An obvious advantage of vertical augmentation is that the surgical shaping of the dental alveolus allows the preparation of an aesthetically pleasing prosthetic work i.e. the teeth do not have to be excessively long to fit the increased vertical dimension. This way, the lips are also supported properly by the teeth, smoothing the wrinkles around the mouth, so the end result makes an even more natural impression [9].

During the reconstructive surgeries, the recipient site was prepared using external incision. In cases where the fibular flap was harvested without a skin paddle, the gingiva of the alveolar process was accessed without incision. In such cases, we consider it important to mobilize the gingiva to the highest possible extent to provide enough space for the flap. This is crucial also because the larger the vertically augmented component, that is, the farther the grafted bone distends from the plane of the floor of the mouth, the easier it is to induce keratinised gingival attachment on the site.

The alveolar process and the teeth support the lips in the frontal region and the cheek in the posterior region [9]. In the frontal region, the lip turns slightly outwards, thereby increasing the visibility of the vermilion border. In cases where the necessary mobilization of the gingiva without intraoral incision was considered infeasible, a fibula flap with a skin paddle was harvested.

The fibula flap was harvested from the donor site using a lateral incision according to Gilbert [10]. Transplantation to the recipient site was carried out according to Hidalgo et al. [11]. The flap was prepared using either a 3 mm muscle cuff or a perforator skin flap. When osteotomy was necessary for the adaption of the bone flap, the procedure was done carefully protecting the periosteal and vascular integrity of the flap. The flap was fastened to the recipient site using mini plates and screws. Suturing on the arterial side was done end-to-end using 8/0 polypropylene suture between the peroneal and the facial or the thyroid arteries. Suturing on the venous side was done either end-to-end between the peroneal and external jugular veins or end-to-side between the peroneal and the internal jugular veins.

The patients spent 10 days in hospital after the surgery. Postoperative antibiotic prophylaxis consisted of amoxicillin with clavulanic acid (1200 mg) and metronidazole (500 mg) iv. three times a day for a week, starting on the day of the surgery.

For each individual case, postoperative follow-up was scheduled at 1, 3, 6 and 12 months after the surgery, and then at 12-month intervals.

### Case 1

**Fig. 1:**
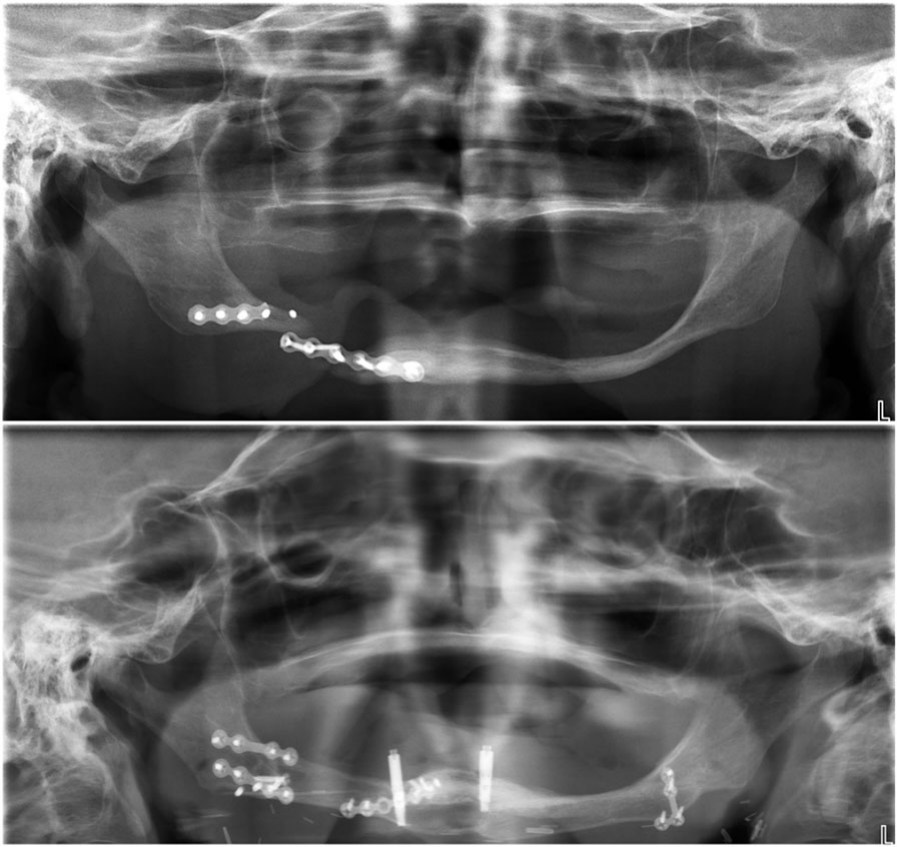
Case 1. Panoramic X-ray images. Top: the preoperative situation. The pathological fracture of the right side of the extremely atrophied mandible with fractured mini plate. The right side was so extremely atrophied in this case that the mandibular bone underwent resorption after the augmentation. Bottom: panoramic X-ray at the 6-year follow-up: the entire body of the mandible was reconstructed with free vascularised fibula flap; dental rehabilitation was provided with two osseointegrated implants

**Fig. 2:**
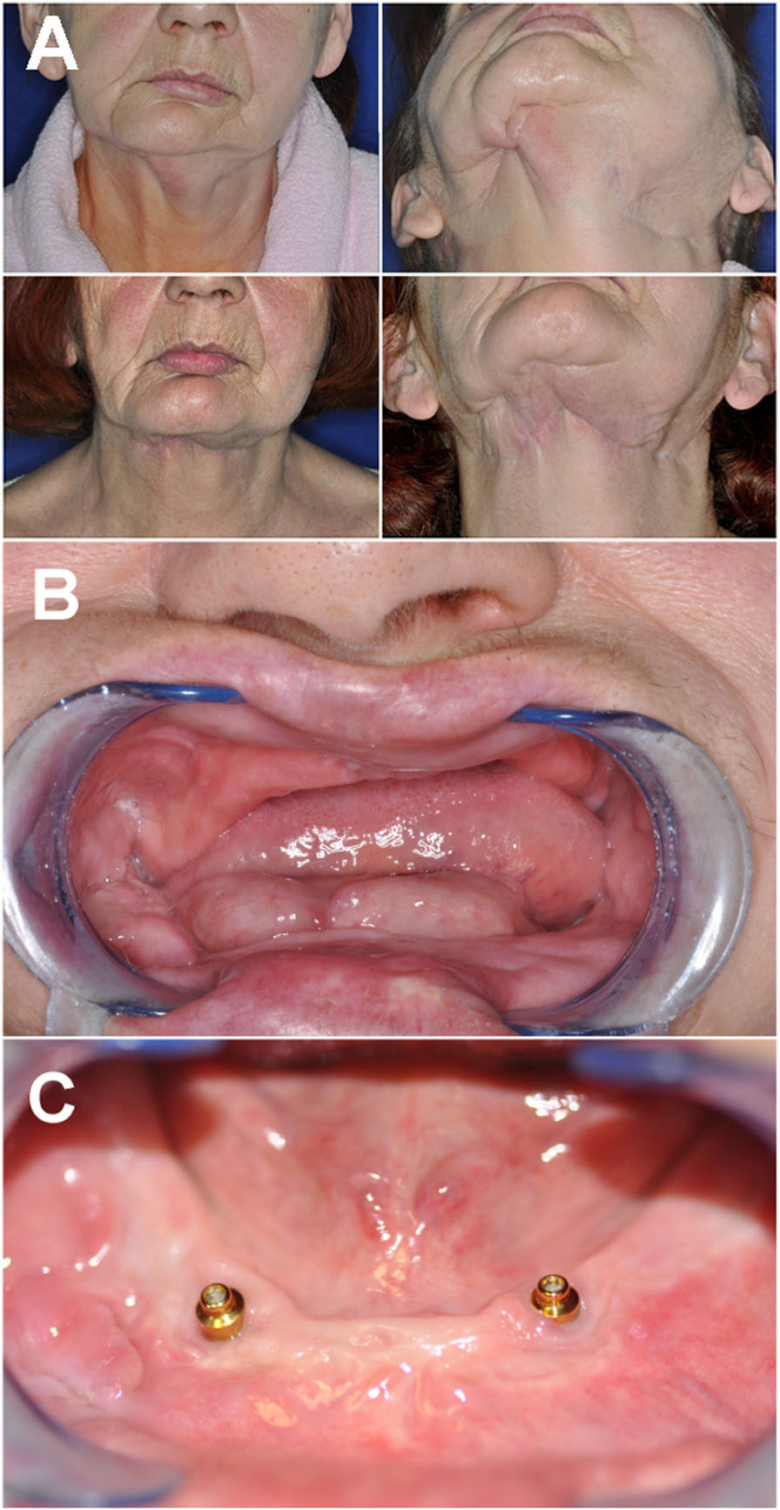
Case 1. Clinical presentation. A: physical presentation before (top) and 6 years after the reconstructive surgery (bottom). The pathological fracture caused marked facial asymmetry (top left) and an orocutaneous fistula had also formed (top right). The surgery eliminated the orocutaneous fistula and brought lasting improvement in the patient’s facial symmetry. B: preoperative status- the floor of the mouth protrudes because of the extreme atrophy and the lack of keratinised gingiva. C: status at the 6-year follow-up. No signs of inflammation or pocket formation around implants

The patient was a 59-year-old female who suffered a pathological fracture caused by the extreme atrophy of the mandible in 2012. Before the pathological fracture, the patient had struggled for altogether 12 years trying to have a properly fitting removable prosthesis made - to no avail, given the extreme atrophy. It turned out from the patient’s history that she had been treated for fracture of the right mandibular angle 11 years before, with miniplate osteosynthesis. Due to the extreme atrophy, though, one of the miniplates had to be completely removed 5 years later, as it had become exposed. The treatment of the pathological fracture (Fig. 1A) was done using free vascularised fibula flap for the vertical augmentation of the entire corpus of the mandible. This way, the risk of repeated fracture was eliminated and implant insertion became possible. The fibula flap was adapted without intraoral incision. The gingiva was mobilised to a great extent to provide enough space for the fibula flap, which is important because the larger the vertically augmented component, the easier it is to gain gingival attachment, especially in the frontal region [12]. The results bore this point out (Fig. 2C). Implantation took place 6 months after the reconstructive surgery. The patient received a locator-retained overdenture.

The panoramic x-ray taken at the 6-year follow-up (Fig. 1 bottom) revealed 2 mm horizontal bone resorption around the implant in the 33 position. No sign of inflammation was observed. The maximum probing depth around the implants was 2 mm and there was no bleeding on probing (Fig. 2C).

### Case 2

**Fig. 3:**
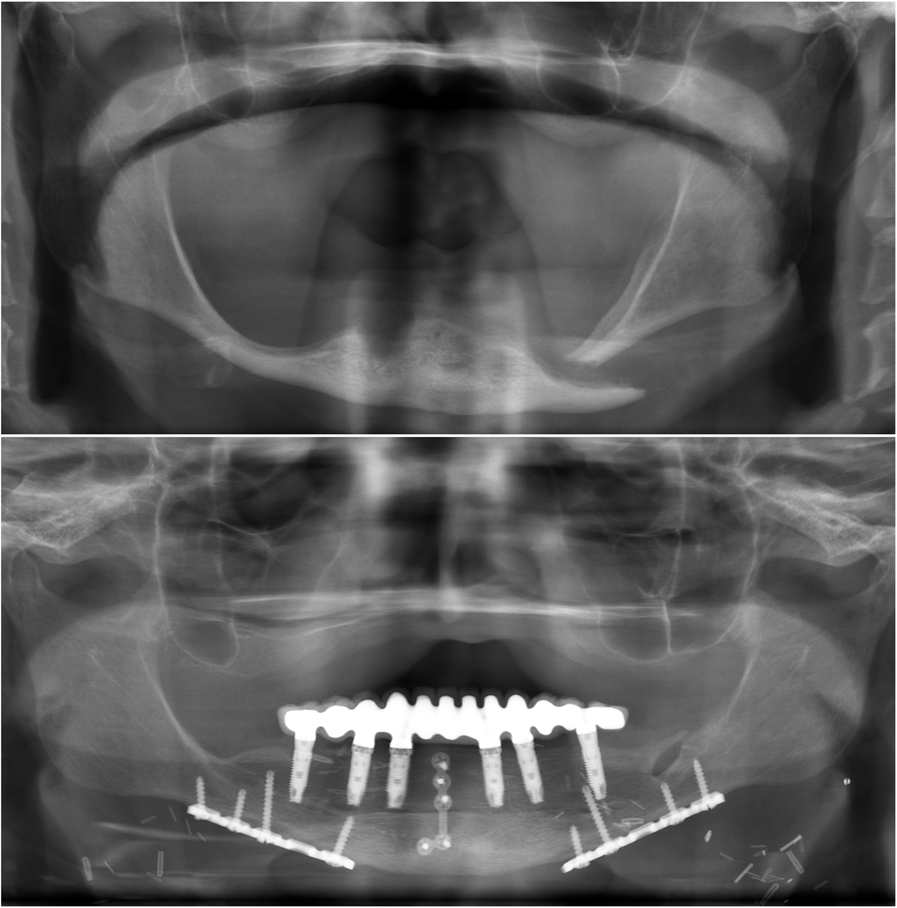
Case 2. Panoramic X-ray images. A: the preoperative situation. The pathological fracture of the left side of the extremely atrophied mandible. B: panoramic X-ray at the 5-year follow-up: the entire body of the mandible was reconstructed with free vascularised fibula flap. The patient received a screw-retained bridge on six osseointegrated implants. No bone resorption was noted around the implant sites

**Fig. 4:**
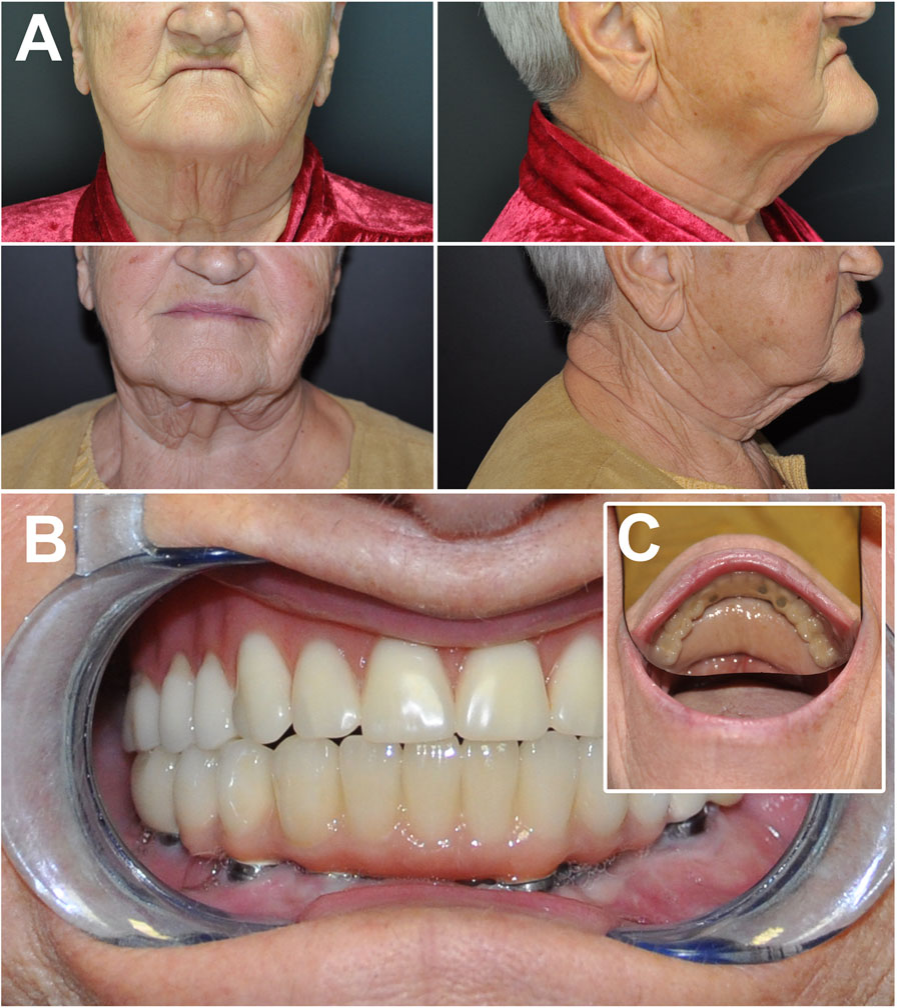
Case 2. Clinical presentation. A: before the surgery (top) and at the 5-year follow-up (bottom). B: The denture at the 5-year follow-up. The buccal aspect of the skin paddle was successfully thinned to an extent that - via the removal of the subcutaneous fat - it was possible to make an esthetically favorable outcome (C shows that the skin paddle is still visible in the floor of the mouth). At the same time, this method yields a sufficient amount of keratinised gingiva

The second case was also a pathological fracture of the severely atrophic mandible (Fig. 3 top). The 68-year-old female patient underwent reconstructive surgery in 2015. We used fibula graft with skin paddle because of the destruction of the mucosal tissue resulting from the compound fracture. Ten months after the surgery, we placed 6 implants and the skin flap was thinned to provide optimal gingival cover. On the 6 implants, we anchored a fixed, screw-retained, full-arch bridge. Similarly to Case 1, this patient had spent one and a half decades prior to the surgery trying to have properly fitting dentures made. Given the high degree of atrophy, all attempts failed. The applied reconstructive treatment restored function and the patient’s facial contours too (Fig. 4).

The panoramic x-ray taken at the 5-year follow-up (Fig. 3 bottom) revealed no bone resorption around the dental implants. Clinical examination confirmed no inflammation. The probing depths around the implants did not exceed 2 mm, and no bleeding on probing was detected.

### Case 3

**Fig. 5:**
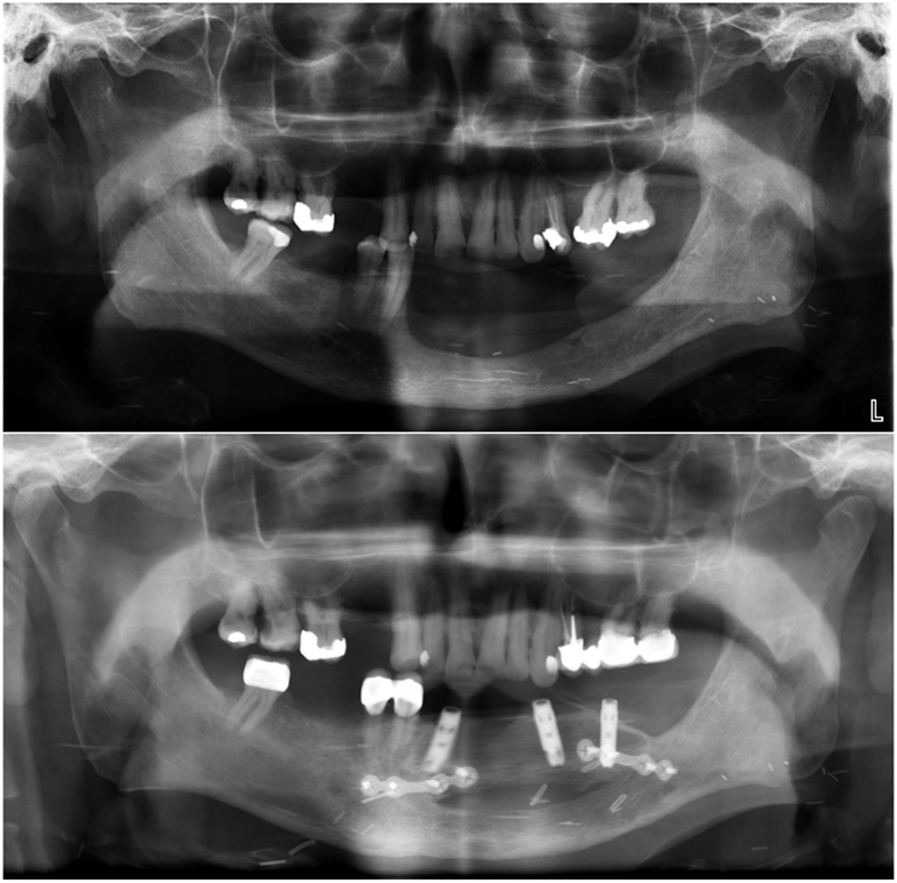
Case 3. Panoramic X-ray images. A: status after the radical cancer surgery and before osseous reconstruction; B: status at the 5-year follow-up: the mandible was reconstructed with free vascularised fibula flap. No bone resorption was observed at the implant sites

**Fig. 6:**
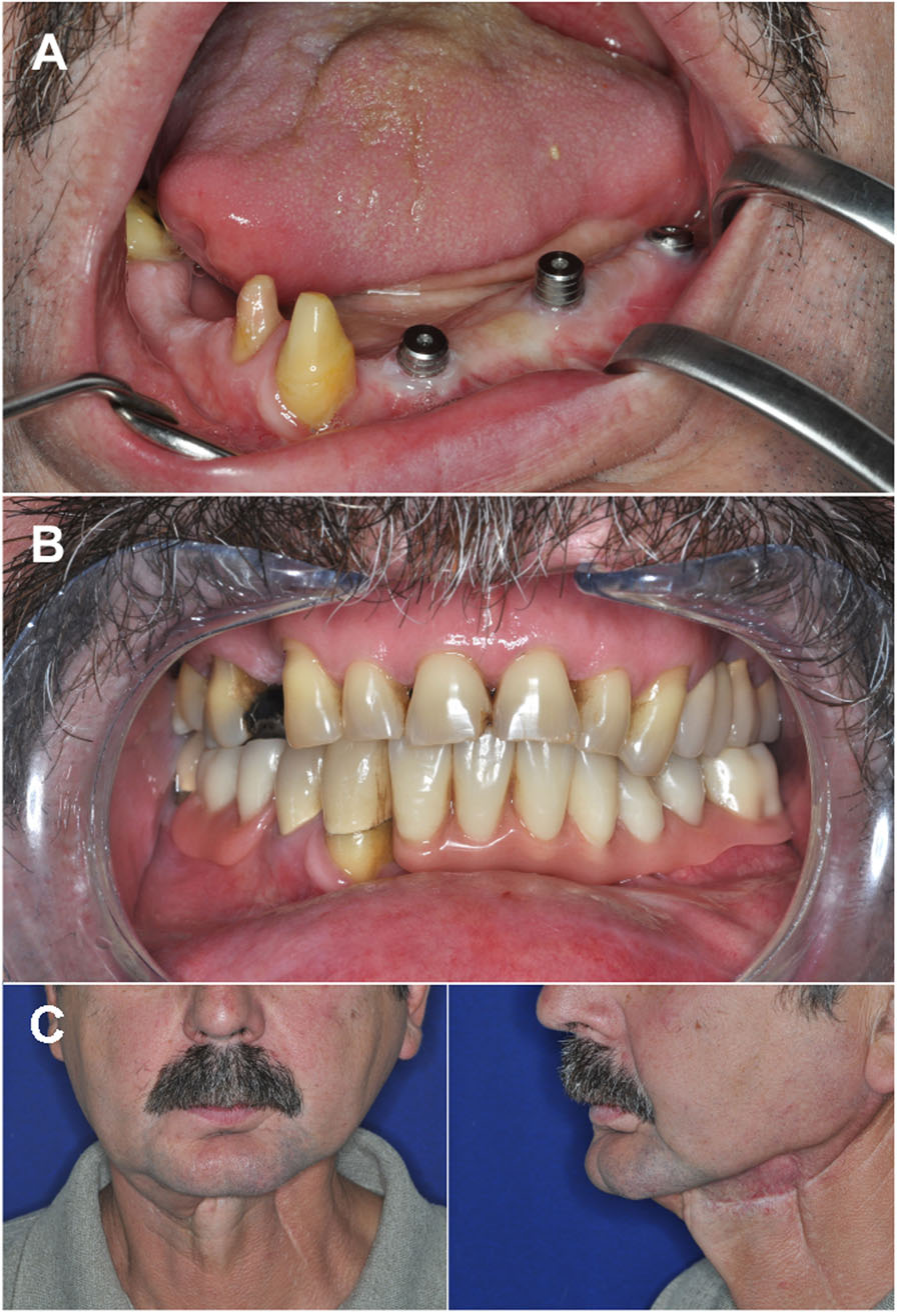
Case 3. Clinical presentation. A: three years after the radical cancer surgery. Clinical picture after gingivoplasty. The implants were surrounded by keratinised gingiva. Neither inflammation nor pocket formation was observed. B and C: clinical presentation at the 5-year follow-up

The fourth case involved reconstructive bone surgery of the left fronto-lateral portion of the mandible (Fig. 5 top). The patient was a 51-year-old male, who had previously undergone radical tumor surgery and soft tissue reconstruction with radial free flap with skin paddle. Two years after the oncological surgery, in 2008, as part of a series of reconstructive surgeries, our aim was to correct the shape of the alveolar ridge to prepare it for implant-based prosthetic treatment. To reach that end, free vascularised fibula flap was used. The skin flap was also corrected to provide optimal gingival cover. This way, we reached near ideal conditions for subsequent dental rehabilitation (Fig. 6). Implant placement was possible 10 months after the surgery. The patient received a locator-retained overdenture. The postoperative radiograph taken at the 5- year follow-up showed no bone resorption around the implants (Fig. 5 bottom) and the clinical examination revealed no sign of inflammation. The probing depths around the implants did not exceed 2.5 mm and no bleeding on probing was detected.

## Discussion and conclusions

In the cases presented here, autologous vertical augmentation with free microvascularised flap has proven to be a reliable approach that allowed lasting esthetic and functional reconstruction. The site healed per primam in all cases. No infection or permanent donor site morbidity was observed. The patients did not report excessive pain, discomfort or any other subjective complaint related to the surgery either in the postoperative or in the follow-up period. The patients’ adherence and compliance was excellent throughout the treatment and also during the follow-up (as indicated by the excellent condition of the dental work and the surrounding hard and soft tissues). The low number of cases can be considered as a weakness of this case series. However, the surgical indication we examined is a relatively rare one, and the long-term outcomes may at least partially make up for that weakness.

Autologous jawbone augmentation methods can be grouped into three main categories, which are as follows: 1) conventional augmentation with intra- or extra-oral autogenous bone grafting; 2) osteodistraction; and 3) free vascularised bone transfer [1, 2]. In our experience, which is in agreement with the literature, the most favorable intraoral donor sites for non-vascularised bone grafting are the mentum and the ramus of the mandible. D1-D2 quality bone can be harvested from both sites [13, 14]. Grafts harvested from these sites are used primarily for lateral augmentation. Bone harvested from the mentum can be used to achieve lateral bone augmentation of 5 mm [15]. Using bone blocks harvested from the ramus region, thinner, 3–4 mm thick D1 quality lateral bone augmentation can be achieved [15, 16]. When it comes to vertical augmentation, though, neither of these methods allow more than 5 mm bone gain [16].

The primary extraoral donor sites are the iliac crest and the calvaria [17]. The main advantage of transplanting bone harvested from the iliac crest is that the site offers a large amount of easily harvestable bone, as well as the simple shaping of the bone blocks [18]. The disadvantage is bone quality: D2-D3 bone is readily resorbed, at rates up to 30–47% [19, 20]. On average, this method yields bone growth of 5–6 mm in both the vertical and horizontal dimensions [21]. The frequency of accompanying donor site morbidity is higher than with other non-vascularised bone grafting modalities. The most common complications are hematoma and seroma, occasionally the paresthesia of the thighs or even the fracture of the iliac spine [21]. For non-vascularised bone transplants, the calvaria is possibly the best donor site for larger vertical augmentation procedures. A large amount of D1 quality bone may be harvested from the calvaria, and the risk of high-degree resorption is low [17, 20, 22]. The thickness of the monocortical bone is 2–3 mm on average, often necessitating the use of a layering technique [20]. On average, this method yields bone growth of 6–7 mm in both the vertical and the horizontal dimensions [23]. Donor site morbidity is low, hematoma and seroma are rare, and post-surgical discomfort is minimal [17, 24].

An entirely different method is osteodistraction, during which the bone is cut and gradually separated, which induces osteogenesis in the resulting gap. This method is used primarily in the reconstruction of vertical deficits. Obvious disadvantages include the discomfort of the patient, the higher risk of infection and that the procedure is time-consuming [25]. However, the results are more reliable, than those obtained using non-vascularised bone transplants: less bone is reabsorbed and higher elevation is possible [15]. It must be noted that the success of this procedure requires an intact bone height of at least 5 mm for the distraction to ensure that the bone is not resorbed or fractured [26]. Free vascularised bone flaps are used primarily in reconstructive surgery, chiefly in the reconstruction of mandibular continuity [5, 27–31]. The procedure is recommended in cases where the mesiodistal extent of the bone defect is more than 5 cm [32]. The native vasculature ensures the survival of the large piece of transplanted bone, which can thus be inserted even into irradiated areas [5]. The transplants used in the reconstruction of major bone defects may also be used in the reconstruction of soft tissue defects when combined with skin paddle relying on septo- or musculocutaneous perforators. The fibula, the iliac crest, the scapula and the radius are the preferred donor sites for the reconstruction of composite maxillofacial defects [30]. The most frequently used donor site is the fibula [5, 27–29], where D1 bone can be harvested. The bone harvested from this site can be 22–25 cm in length and may be used for vertical augmentation of 1–1.5 cm height, which makes fibula graft extremely suitable for the reconstruction of mandibular defects [28, 33]. As for the free vascular flaps, the iliac crest is also suitable for grafting as it is easy to shape and model and can be used to achieve significant vertical augmentation on segments up to 7–9 cm in length. However, the density of the bone is significantly lower D2–3 and donor site morbidity is higher compared to fibular harvest [34]. The radial free flap can be used for vertical bone augmentation of 5-7 mm on segments 8–12 cm in length [33]. Considering the risk of donor site fracture, prophylactic plating of the radius is recommended. The scapular flap is suitable for the reconstruction of bone defects up to 10–15 cm in length, but it offers less bone in the vertical dimension than the free iliac [35] or fibula [36] flap.

The disadvantages of microsurgical reconstructive methods include the necessity of special training and equipment, the risk of donor site morbidity and that these procedures are time-consuming [32]. In the presented cases, mandibular continuity was maintained with a vertical bone defect of at least 1 cm over a segment of at least 5 cm, and the distance between the inferior alveolar nerve or the base of the mandible and the alveolar ridge was smaller than 5 mm. That is, these cases were definitely in need of extensive vertical augmentation. Taking this into consideration, we opted for microsurgical mandibular reconstruction in all cases. According to the literature, such procedures have excellent success rates [5, 27–31]. However, there are two noteworthy disadvantages. First, the microsurgical operation is lengthy, which means that the patient is exposed to prolonged general anesthesia. Second, while the chance of morbidity is low indeed, it may be more severe as compared to other method. Donor site complications may include sensory loss, ankle instability, or contracture of the great toe [37]. Still, we argue that the advantages outweigh the disadvantages. As demonstrated in the presented cases, this method makes it possible to reconstruct large defects, even in irradiated areas. When combined with a skin paddle, the fibula free flap may be used for the reconstruction of soft tissue defects, and it allows optimal gingival coverage for implant and prosthetic procedures. Finally, it leads to immediate aesthetic results, as shown by our cases and by other published cases [5, 27–31]. Therefore, we recommend the described approach for the treatment of large mandibular defects with maintained continuity.

## Data Availability

The datasets generated and/or analysed during the current study are not publicly available due to protecting individual patient privacy but are available from the corresponding author on reasonable request.
